# Short‐Course Blinatumomab Treatment as a Bridge to Further Salvage Therapy for Relapsed/Refractory B‐Cell Acute Lymphoblastic Leukemia: A Retrospective Single‐Center Study

**DOI:** 10.1002/cam4.70515

**Published:** 2024-12-18

**Authors:** Jin Yin, Xiaoya Cai, Bingxin Qian, Ying Liu, Dengju Li

**Affiliations:** ^1^ Department of Hematology, Tongji Hospital, Tongji Medical College Huazhong University of Science and Technology Wuhan China

**Keywords:** B‐cell acute lymphoblastic leukemia, blinatumomab, relapsed/refractory, short‐course

## Abstract

**Backgroud:**

The high cost of blinatumomab in full doses of full treatments has led to dose reduction and fewer treatment cycles for most patients in China. With current needs for cost‐efficiency and resource management in health care, we retrospectively evaluated the clinical effects of short‐course blinatumomab treatment for R/R Ph‐ B‐ALL at our center.

**Methods:**

Blinatumomab was administered with 24‐h continuous intravenous infusion (9 μg/day for the first 3 days and 28 μg/day for 6–10 days). The clinical data of 30 R/R B‐ALL patients were collected and analyzed.

**Results:**

A total of 25 patients (83.3%) including 13 (43.3%) with a high leukemic load (> 50%) achieved morphological CR. Twelve patients (40%) were MRD‐negative. The estimated 2‐year OS rate was 82.62%. The 2‐year PFS rate was 78.35%. The estimated 2‐year OS and PFS were significantly better in patients receiving further treatment.

**Conclusions:**

Our findings provide novel insights into the optimization of blinatumomab therapy, proposing a viable treatment alternative that aligns with current needs for cost‐efficiency and resource management in health care.

## 1 Introduction

Although the use of pediatric‐like or pediatric‐inspired protocols in adults with Philadelphia chromosome‐negative (Ph‐) B‐cell acute lymphoblastic leukemia (B‐ALL) allowed markedly improving the outcome of young adults aged up from 40 to 60 years, with estimated 5‐year overall survival (OS) comprised between 60% and 70% [1, 2, 3, 4], long‐term survival remains poor (40%–50%) [5, 6, 7]. For patients with relapsed/refractory (R/R) B‐ALL, complete remission (CR) rates range from 20% to 30%, with 3‐year overall survival (OS) varies from 6% to 26% [8, 9, 10, 11]. Allogeneic hematopoietic stem cell transplantation (allo‐HSCT) remains a long‐term curative option for R/R B‐ALL patients. However, for those considered unfit for allo‐HSCT, either owing to lack of remission or health condition, the primary challenge is safely bridging patients to subsequent treatments to obtain more chances of achieving CR, thereby getting the opportunity for allo‐HSCT and achieving long‐term survival.

Blinatumomab is a CD19 × CD3 bispecific T‐cell engager (BiTE) antibody. Studies demonstrate significant clinical benefits of blinatumomab in both minimal residual disease (MRD)‐positive B‐ALL and R/R B‐ALL, including higher overall response rates, enhanced 6‐month progression‐free survival (PFS), and improved median OS, compared with those receiving chemotherapy [12, 13, 14]. However, the high cost of blinatumomab treatment has led to dose reductions and a decrease in the number of treatment cycles for a significant number of R/R patients in China. Therefore, we conducted this study to retrospectively assess the clinical benefit of short‐course blinatumomab treatment in patients with R/R Philadelphia chromosome‐negative (Ph‐) B‐ALL.

## 2 Patients and Methods

### 2.1 Study Design

We retrospectively collected data from electronic medical records of adult patients with R/R Ph‐ B‐ALL who received short‐course blinatumomab between 2021 and 2022 at our institution. The study was conducted in accordance with the Declaration of Helsinki principles and approved by the Medical Ethics Committee of Tongji hospital.

### 2.2 Treatment

The short‐course was defined in this study as full dosage blinatumomab treatment used for less than 10 days, excluding dose escalation period. Blinatumomab was administered with 24‐h continuous intravenous infusion (9 μg/day for the first 3 days and 28 μg/day for 6–10 days). Corticosteroid pretreatment was mandatory for the prophylaxis of neurological events and cytokine release syndrome (CRS) during blinatumomab treatment.

### 2.3 Assessment of Remission

Remission was subclassified depending on peripheral blood count recovery into CR with full hematologic recovery (CR: Bone marrow blasts < 5%; absence of circulating blasts or blasts with Auer rods; absence of extramedullary disease; ANC ≥ 1.0 × 10^9^ /L; platelet count ≥ 100 × 10^9^/L) and CR with incomplete hematologic recovery (CRi; All CR criteria except for residual neutropenia < 1.0 × 10^9^/L or thrombocytopenia < 100 × 10^9^/L). The definition of ‌Composite complete response (cCR)‌ includes ‌CR‌ or ‌CRi‌. Complete molecular remission was assessed using real‐time quantitative polymerase chain reaction (RT‐QPCR) for leukemia‐specific rearrangements of immunoglobulin genes and other genetic alterations. MRD levels < 0.01% (< 10^−4^) were considered negative. Relapse was defined by the reappearance of over 5% blasts in the bone marrow or by the presence of extramedullary disease post‐CR, including central nervous system (CNS) relapse. Progression was defined by a non‐CR response postblinatumomab treatment or the presence of extramedullary disease concurrent with the achievement of CR. Bone marrow (BM) was evaluated prior to the initial blinatumomab treatment and postrecovery from myelosuppression (neutrophil count > 1000/mL); in patients without myelosuppression, BM was evaluated on Day 14 postblinatumomab infusion cessation.

### 2.4 Statistical Analysis

OS was defined as the time from Day 1 of the first blinatumomab cycle to death from any cause or the last follow‐up. PFS was defined as the time from Day 1 of the first blinatumomab cycle to the earliest indication of disease progression (including objective progression and relapse from CR), death from any cause, or last follow‐up. For nonresponders, PFS defined as the time from Day 1 of the first blinatumomab cycle to the day of first BM assessment. Adverse events were graded according to the Common Terminology Criteria for Adverse Events (CTCAE) v5.0. Statistical analyses were performed using SPSS Statistics (version 25.0; IBM Corp., Armonk, NY, USA) and GraphPad Prism 9 (GraphPad Software Inc., CA, USA). Data are presented as descriptive values, percentages, medians, and ranges, as appropriate. Survival data were estimated using the Kaplan–Meier method.

## 3 Results

### 3.1 Patient Characteristics

Thirty patients with R/R Ph– B‐ALL received short‐course blinatumomab treatment between 2021 and 2022 at our institution. The median age was 38 years (range, 18–77 years). This study included 14 men and 16 women. Most patients (21/30) experienced relapse, whereas nine patients with primary refractory disease showed no response to second‐line or additional therapies. Based on genetic aberrations, 17 patients were classified as standard‐risk and 13 as poor‐risk [15]. None of the patients had CNS involvement or extramedullary disease. Patient characteristics are presented in Table 1.

**TABLE 1:** Patient characteristics.

Patients	Age (years)	Gender	Disease status at the time of blina	Prognostic risk genetic aberration	Preblina therapy
1	52	M	Primary refractory	Poor	Chemotherapy
2	64	F	2R	Standard	Chemotherapy
3	18	F	Primary refractory	Standard	Chemotherapy
4	32	M	Primary refractory	Standard	Chemotherapy
5	41	F	1R	Standard	Chemotherapy
6	18	M	3R	Standard	Chemotherapy
7	61	F	2R	Standard	Chemotherapy
8	46	F	3R	Poor	Chemotherapy+allo‐ HSCT
9	27	F	3R	Standard	Chemotherapy
10	32	F	1R	Poor	Chemotherapy
11	52	M	Primary refractory	Poor	Chemotherapy
12	35	F	1R	Standard	Chemotherapy
13	61	F	1R	Standard	Chemotherapy
14	27	M	2R	Standard	Chemotherapy
15	20	M	1R	Poor	Chemotherapy+allo‐ HSCT
16	42	M	Primary refractory	Standard	Chemotherapy
17	22	F	1R	Poor	Chemotherapy
18	34	F	1R	Standard	Chemotherapy+allo‐ HSCT
19	32	M	1R	Poor	Chemotherapy
20	30	F	Primary refractory	Poor	Chemotherapy
21	28	M	Primary refractory	Poor	Chemotherapy
22	57	F	Primary refractory	Poor	Chemotherapy
23	22	M	2R	Standard	Chemotherapy+allo‐HSCT+CART
24	51	M	2R	Poor	Chemotherapy
25	77	F	Primary refractory	Standard	Chemotherapy
26	19	F	1R	Standard	Chemotherapy
27	32	F	1R	Standard	Chemotherapy
28	54	M	1R	Standard	Chemotherapy
29	33	M	1R	Poor	Chemotherapy
30	39	M	2R	Poor	Chemotherapy

*Note:* Before the treatment of blinatumomab, cases 8, 15, 18 relapsed after allo‐ HSCT. Case 23 experienced the first relapse after allo‐HSCT and a second relapse after CD19/CD22 CAR‐T treatment.

Abbreviations: Blina, blinatumomab; BM, bone marrow; F, female; allo‐ HSCT, allogeneic hematopoietic stem cell transplantation; M, male; 1R, the first relapse; 2R, the second relapse; 3R, the third relapse.

### 3.2 Outcomes

The median BM blast level before blinatumomab therapy was 49.06% (range: 6%–99%). Twenty‐five patients (83.3%) achieved morphological CR (CR *n* = 16, CRi *n* = 9), and 12 (40%) reached MRD negativity. Thirteen patients (43.3%) had a high leukemic load (> 50%) prior to blinatumomab therapy. Nine (69.2%) of these patients, including six (46.2%) achieving MRD negativity, responded to blinatumomab. Five patients (38.5%) showed no response, including one primary refractory patient, two with a third relapse, one with a second relapse, and one with a first relapse. All five nonresponders died (two after receiving CAR‐T therapy, one from relapse, and another from infection). Among the 25 responders (83.3%), three (12.0%) underwent no further treatment and experienced no relapse by the end of the follow‐up. Twenty‐two (88.0%) received further treatment, including chemotherapy (*n* = 8), additional cycles of short‐course blinatumomab (*n* = 3), CAR‐T therapy (*n* = 3), and allo‐HSCT (*n* = 8). By the end of follow‐up, five (22.7%) relapsed (three patients with chemotherapy, one patient with allo‐HSCT, and one patient with CAR‐T therapy). Four of these patients received additional treatment, whereas one died owing to treatment withdrawal. Details are presented in Table 2.

**TABLE 2:** Clinical efficacy of blinatumomab.

Patients	Disease status at the time of blina	BM blast% at onset of blina	Prognostic risk genetic aberration	Response to blina	MRD‐negative	Follow‐up therapy after blina	Relapse	Cause of death
1	Primary refractory	31	Poor	CR	Yes	Blina	No	Alive
2	2R	6	Standard	CRi	No	Allo‐HSCT	No	Alive
3	Primary refractory	29	Standard	CRi	No	CART+allo‐HSCT	No	Alive
4	Primary refractory	12	Standard	CRi	No	Chemotherapy	No	Alive
5	1R	27	Standard	CR	Yes	CART	No	Alive
6	3R	99	Standard	No‐response	No	CART	No	Infection
7	2R	35	Standard	CR	Yes	Chemotherapy	Yes	Alive
8	3R	66	Poor	CRi	No	Chemotherapy	No	Alive
9	3R	82.5	Standard	No‐response	No	No	—	Leukemia
10	1R	43	Poor	CR	No	Chemotherapy	No	Alive
11	Primary refractory	98	Poor	No‐response	No	No	—	Leukemia
12	1R	71	Standard	CR	Yes	Chemotherapy	No	Alive
13	1R	54	Standard	CRi	Yes	Chemotherapy	Yes	Leukemia
14	2R	33	Standard	CR	No	Allo‐HSCT	No	Alive
15	1R	81	Poor	CR	Yes	No	No	Alive
16	Primary refractory	30	Standard	CR	Yes	No	No	Alive
17	1R	64.5	Poor	CRi	No	CART	Yes	Alive
18	1R	92	Standard	CR	Yes	CART	Yes	Alive
19	1R	11	Poor	CR	Yes	No	No	Alive
20	Primary refractory	17	Poor	CR	Yes	Allo‐HSCT	No	Alive
21	Primary refractory	6.5	Poor	CR	No	Allo‐HSCT	Yes	Alive
22	Primary refractory	88	Poor	CR	Yes	Allo‐HSCT	No	Alive
23	2R	62	Standard	CR	Yes	Allo‐HSCT	No	Alive
24	2R	94	Poor	No‐response	No	No	—	Leukemia
25	Primary refractory	9	Standard	CRi	No	Blina	No	Alive
26	1R	6	Standard	No‐response	No	CART	—	Leukemia
27	1R	10.5	Standard	CRi	No	Allo‐HSCT	No	Alive
28	1R	38	Standard	CRi	No	Chemotherapy	No	Alive
29	1R	92	Poor	CR	No	Blina	No	Alive
30	2R	7	Poor	CR	No	Chemotherapy	No	Alive

*Note:* CR, bone marrow blasts < 5%; absence of circulating blasts or blasts with Auer rods; absence of extramedullary disease; ANC ≥ 1.0 × 10^9^ /L; platelet count ≥ 100 × 10^9^/L; CRi; all CR criteria except for residual neutropenia < 1.0 × 10^9^/L or thrombocytopenia < 100 × 10^9^/L.

Abbreviations: Allo‐HSCT, allogeneic hematopoietic stem cell transplantation; blina, blinatumomab; CART, CD19/CD22 CAR‐T.

### 3.3 Toxicity of Short‐Course Blinatumomab Therapy

No infusion‐related reactions, neurological events, or elevated liver enzymes were observed. Over half of the patients (25/30, 83.8%) experienced hematological toxicities. Grade ≥ 3 AEs occurred in 11 patients with neutropenia (36.7%), nine with anemia (30%), and 11 with thrombocytopenia (36.7%). Other major side effects included CRS (*n* = 17, 56.7%), infection (*n* = 4, 13.3%), and tumor lysis syndrome (*n* = 2, 6.7%). Grade 3 or 4 CRS was observed in four patients. No treatment‐related deaths occurred during the study period (Table 3).

**TABLE 3:** Adverse events during blinatumomab treatment.

Adverse event	Number of patients (*N* = 30)	
	Any grade	Grade ≥ 3
Neutropenia	22	11
Anemia	25	9
Thrombocytopenia	19	11
Fever	17	5
Infection	4	0
Cytokine release syndrome	17	4
Tumor lysis syndrome	2	0
Capillary leak syndrome	0	0
Neurologic event	0	0
Elevated liver enzymes	0	0
Infusion‐related reaction	0	0

### 3.4 Survival

**FIGURE 1:**
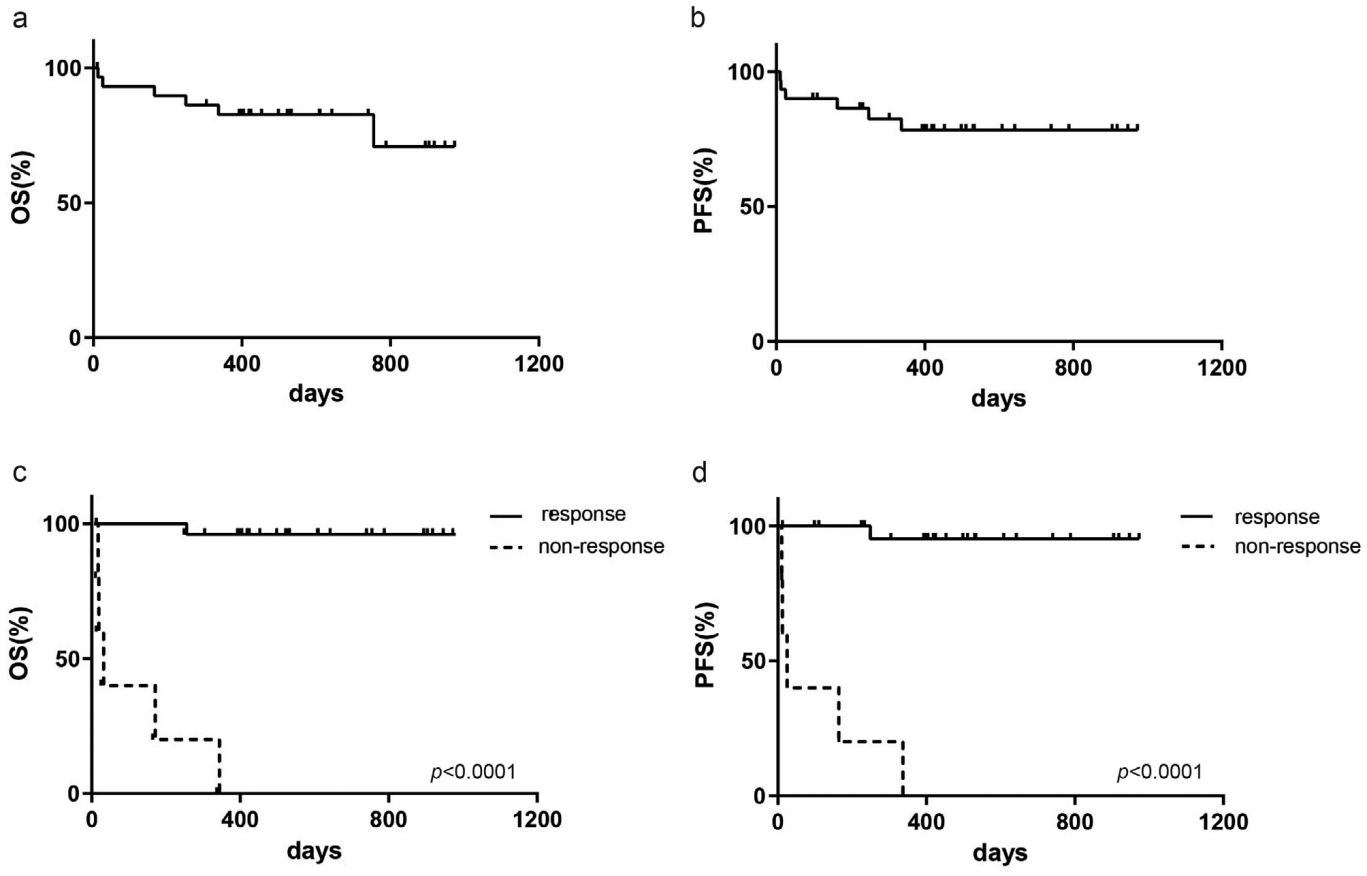
Clinical outcomes for patients after short‐course blinatumomab treatment. (a, b) The 2‐year OS and PFS for all patients in this study. (c, d) Patients who had response to blinatumomab had better 2‐year OS and PFS.

**FIGURE 2:**
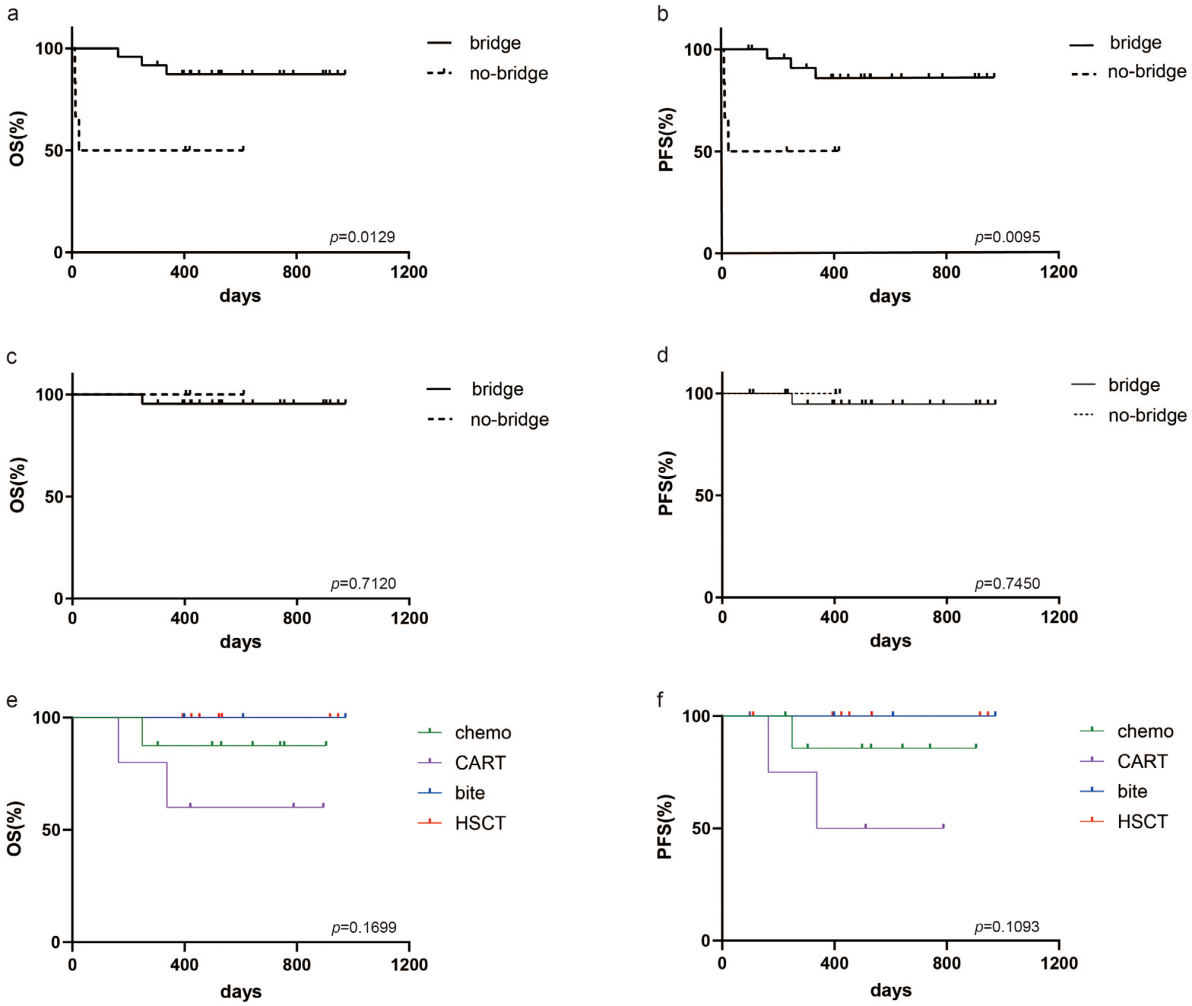
Clinical outcomes for patients with further treatment post blinatumomab. (a, b) Patients with further treatment post blinatumomab showed better 2‐year OS and PFS. (c, d) Responders to blinatumomab receiving further treatments post blinatumomab, 2‐year OS and PFS had not differ significantly. (e, f) Comparative analysis among different therapies (chemotherapy, CAR‐T thearpy, HSCT, and blinatumomab groups) postblinatumomab in responders indicated no differences in 2‐year OS and PFS.

By the end of the follow‐up period (April 30, 2024), the median follow‐up was 17 months (range 1–32.4 months). For all patients, the estimated 2‐year OS rate was 82.62% (95%CI 63.14–92.37). The 2‐year PFS rate was 78.35% (95%CI 57.79–89.71) (Figure 1a,b). Median OS was not defined. Responders to blinatumomab therapy had a 2‐year OS rate of 96.0% (95%CI 74.84–99.43, *p* < 0.0001) and 2‐year PFS 95.24% (95%CI 70.72–99.32, *p* < 0.0001), whereas nonresponders experienced poor outcomes (2‐year OS 0% and 2‐year PFS 0%). The 6‐month survival rate for nonresponders was 20% (95%CI 0.84–58.19), and all nonresponders died within 1 year of starting blinatumomab treatment (Figure 1c,d). Patients who received further treatment post‐blinatumomab exhibited better 2‐year OS (87.30%, 95%CI 65.58–95.72 vs. 50.0% 95%CI 11.10–95.72, *p* = 0.0129) and PFS (85.64% 95%CI 61.72–95.14 vs. 50.0% 95%CI 11.10–80.37, *p* = 0.0095) compared with those who did not (Figure 2a,b). The median OS and PFS for patients without further treatment were 10.5 and 7.4 months, respectively. Median OS and PFS were undefined in patients with further treatment. The 2‐year OS (100% vs. 95.46%, *p* = 0.712) and PFS (100% vs. 94.74%, *p* = 0.745) between responding patients with and without further treatment did not differ significantly (Figure 2c,d). Among different further treatments (chemotherapy, CAR‐T, HSCT, and blinatumomab groups) for patients in this study, comparative analysis indicated no differences in 2‐year OS and PFS (Figure 2e,f).

## 4 Discussion

Since blinatumomab became available for B‐ALL, numerous studies have been conducted to compare the efficacy of blinatumomab as a single agent against traditional salvage chemotherapy in R/R Ph+ and Ph– B‐ALL, demonstrating significant improvements in remission rates.Owing to its substantially short half‐life, blinatumomab requires administration as a 24‐h continuous intravenous infusion, typically over 4 weeks with a 2‐week rest period between cycles. Patients treated with blinatumomab received up to five cycles of induction and consolidation, followed by maintenance blinatumomab for up to 1 year [13, 14, 16, 17, 18]. With current needs for cost‐efficiency and resource management in health care, we retrospectively evaluated the clinical effects of short‐course blinatumomab treatment for R/R Ph‐ B‐ALL at our center.

The short‐course was defined in this study as full dosage blinatumomab treatment used for less than 10 days, excluding dose escalation period. Given the cost constraints, we have shortened the time for dose escalation. Our study showed that shortening the blinatumomab infusion time to 3 days at a dose of 9 μg/day was safe. Rapid dose accumulation in the first 3 days did not increase drug toxicity. In this study, the cCR rate (83.3%) was higher than expected (approximately 50%) based on previously reported clinical trials (34%–43%) and 40% achieved MRD‐negative [13, 16]. According to real‐world studies of patients with R/R disease treated with blinatumomab outside the context of clinical trials, the CR rate ranged from 44.9% to 80.8% and 44% achieved CR MRD‐negative [19, 20, 21, 22, 23]. In our center, the cCR rates of short‐course therapy were consistent with real‐world data reported for standard blinatumomab treatment. We speculated that rapid dose accumulation and full dosage maintenance in the early stages of treatment were critical factors in reducing tumor load and achieving CR quickly. Although four of the five nonresponding patients had substantially high levels of leukemia blasts, we hypothesized that non‐responding patients did not benefit from 28 days of blinatumomab treatment after 7–10 days.

Several factors have been identified that affect response and a high disease burden (> 15% of leukemic blasts) is reportedly the primary cause [24, 25, 26]. In patients with low‐burden disease, blinatumomab has resulted in long‐term survival in the absence of further therapy [24, 25]. In this study, none of the patients showed loss of CD19 expression and none of the patients with multilinear recurrence achieved MRD negativity. Three of the five nonresponding patients had multilinear recurrence. We hypothesized that patients with multilinear recurrence required a longer treatment time than the short‐course treatment to achieve MRD negativity compared with that in primary refractory and first‐relapsed patients. However, this is the first study to propose a short‐course blinatumomab treatment strategy. Comparative studies of the treatment efficacy between short‐ and standard‐course blinatumomab are warranted to provide additional clinical evidence.

In this study, 88% of responding patients and 40% of nonresponding patients received further salvage therapy. The estimated 2‐year OS and PFS were significantly better in patients receiving further treatment. Although the median OS and PFS in all patients were not defined in this study, the median OS of patients without further treatment after blinatumomab was 7.4 months, which is close to the 7.7 months reported by the Dr. Kantarjian study, in which the majority of patients in that study experienced their first or second relapse [14]. All clinical outcomes in our study indicated that short‐course blinatumomab could significantly benefit patients with R/R Ph‐ B‐ALL. The therapeutic efficacy at 2 years was not inferior to the results of 28‐day administration in terms of remission rates and clinical outcomes at 2 years [13, 14, 16, 17, 18]. No significant difference was observed in 2‐year OS and PFS among responding patients receiving chemotherapy, CAR‐T therapy, and allo‐HSCT as further treatment strategies. By the end of the follow‐up period, 20% of the responding patients had relapsed, with only one patient relapsing after allo‐HSCT. Short‐course blinatumomab bridging to allo‐HSCT seems to have better clinical outcomes. However, owing to the limited follow‐up time and number of cases, updated survival data will be needed in the future to validate our speculation. Our study had the following limitations: the number of patients was limited, and prospective studies are necessary to evaluate the treatment efficacy of short‐course versus standard‐course blinatumomab treatment strategies in the future. Based on our clinical results in this study, the registered clinical trial has been performed in our center aimed to evaluate the clinical outcomes between short‐course treatment and 28‐day treatment of blinatumomab in R/R B‐ALL patients.

## 5 Conclusion

In conclusion, a short course of blinatumomab was considered a bridge to further salvage therapy aimed at achieving disease remission and reducing treatment costs. Notably, our findings provide novel insights into the optimization of blinatumomab therapy, proposing a viable treatment alternative that aligns with current needs for cost‐efficiency and resource management in healthcare.

## Author Contributions

Xiaoya Cai, Bingxin Qian, Ying Liu and Jin Yin contributed toward data collection, data analysis and data interpretation. Jin Yin contributed toward preparation of figures, and writing of the manuscript. Dengju Li design the study. All authors had full access to all the data in the study and accept responsibility to submit for publication.

## Ethics Statement

The study design was approved by the Medical Ethics Committee of Tongji hospital and conducted in adherence to the Declaration of Helsinki.

## Consent

The need for informed consent was waived owing to the retrospective nature of the study.

## Conflicts of Interest

The authors declare no conflicts of interest.

## Data Availability

The additional data collected in this study are available from the corresponding authors upon reasonable request.
